# Advancing Stable Isotope Analysis for Alcoholic Beverages’ Authenticity: Novel Approaches in Fraud Detection and Traceability

**DOI:** 10.3390/foods14060943

**Published:** 2025-03-10

**Authors:** Yiqian Ma, Yalan Li, Feilong Shao, Yuanyu Lu, Wangni Meng, Karyne M. Rogers, Di Sun, Hao Wu, Xiaodong Peng

**Affiliations:** 1 Guizhou Institute of Products Quality Inspection & Testing, Guiyang 550016, China; m13984883094_1@163.com (Y.M.); dragonshao1986@163.com (F.S.); 18786639226@163.com (Y.L.); mengwangni406@163.com (W.M.); 15285982595.520@163.com (X.P.); 2 Key Laboratory of the Ministry of Education for Coastal and Wetland Ecosystems, College of the Environment and Ecology, Xiamen University, Xiamen 361102, China; liyalan7@163.com; 3 National Isotope Centre, GNS Science, Lower Hutt 5040, New Zealand; k.rogers@gns.cri.nz

**Keywords:** stable isotope analysis, alcoholic beverages, authenticity, chemometric modeling

## Abstract

Background: Alcoholic beverages have been popular for thousands of years due to their unique flavors and cultural significance. However, the industry’s high profit margins have led to increasingly sophisticated counterfeiting practices. Stable isotope analysis has emerged as one of the most promising techniques for addressing authenticity and traceability challenges in alcoholic beverages. Scope and approach: This review presents a comprehensive summary of the principles and recent advancements in the application of stable isotope techniques for authenticity assessment. It examines their use in detecting fraud (e.g., identifying edible alcohol, exogenous water, carbonylation, and trace compounds), vintage identification, and geographical origin determination across various alcoholic beverages, with a particular focus on wine, Chinese baijiu, and beer. Conclusions: Stable isotope analysis is a powerful tool for verifying the authenticity of alcoholic beverages, offering effective solutions to combat counterfeiting, mislabeling, and adulteration. Future studies should focus on understanding the ecological, biological, and hydrometeorological factors influencing isotope signatures and develop advanced multi-isotope and chemometric approaches to improve reliability. Expanding global databases and integrating emerging technologies such as artificial intelligence (AI) and machine learning will further enhance the effectiveness and accessibility of stable isotope techniques, ensuring safer and higher-quality alcoholic beverages for consumers worldwide.

## 1. Introduction

Alcoholic beverages, defined as drinks with an ethanol content of 0.5% vol or higher, have been consumed globally for centuries valued for their cultural significance and diverse flavors. However, the rapid globalization of the alcoholic beverages market has introduced significant challenges, such as counterfeiting, adulteration, and mislabeling. These issues not only pose risks to consumer safety but also threaten market integrity [1,2,3,4,5,6,7]. The authenticity of alcoholic beverages has thus become a crucial concern, as it ensures that consumers can purchase genuine products, protects producers’ rights to geographical origin labeling and legal production, and provides essential technical support for market supervision. Addressing these challenges requires reliable methods to ensure the authenticity and traceability of alcoholic beverages, thereby safeguarding public health and preserving industry standards.

To solve the problem of alcoholic beverage counterfeiting, various technological tools have been developed to identify the adulteration of alcoholic beverages. The most commonly used techniques include spectroscopic techniques [8,9], chromatography and their combination techniques [10], and stable isotope techniques [11]. Among the existing analytical techniques, stable isotope techniques have emerged as one of the most promising methods for authenticity verification. Unlike molecular-level analyses such as spectroscopy or chromatography, stable isotope technology operates at the atomic level [12], offering the advantage of isotope ratios that remain stable despite exogenous additives. These ratios encode valuable information on geographic origin, production processes, and vintage, making them a powerful tool for addressing the growing complexity of authenticity issues [13].

Stable isotopes are categorized into light bio-elements (e.g., carbon, hydrogen, oxygen, and nitrogen) and heavy geo-elements (e.g., strontium, lead) [14,15]. Light bio-elements are often used in authenticity studies due to their stable isotope ratios, while geo-elements, including heavy isotope variations, provide additional information on geographical origin. Techniques such as isotope ratio mass spectrometry (IRMS) or multi-collector inductively coupled plasma mass spectrometry (MC-ICP-MS) enable precise quantification of these isotopes in alcoholic beverages. The composition of stable isotopes is commonly expressed in terms of *δ* values, which indicate the deviation in parts per thousand of the stable isotope ratios of an element in a sample relative to the corresponding ratio in a standard substance, calculated by Equation (1), and normalized with that of the international standard [12]. Where *δ* represents the ratio between the heavy and light isotopes, and *R*_sample_ and *R*_standard_ are the isotope ratios (such as ^2^H/^1^H, ^1^⁸O/^1^⁶O, or ^13^C/^12^C) in the sample and the reference material, respectively.

*δ* (‰) = (*R*_sample/_*R*_standard_ − 1) (1)

The carbon (*δ*^13^C), hydrogen (*δ*^2^H), oxygen (*δ*^18^O), and strontium (^87^Sr/^86^Sr) stable isotopes are the most commonly used indicators in authenticity research of alcoholic beverages, and different stable isotopes have different fractionation mechanisms. For instance, the fractionation of carbon isotopes in alcohols, as reflected in *δ*^13^C values, is collectively influenced by photosynthesis, biological metabolism, fermentation, and distillation processes. These processes affect the carbon isotopic composition through their impact on the isotopic characteristics of raw materials, the metabolic pathways of microorganisms, the isotopic fractionation of fermentation products, and the physicochemical transformations occurring during distillation [16,17,18]. Hydrogen and oxygen isotopes are mainly influenced by physical fractionation during evaporation, water absorption by plants, fermentation process, and distillation process, where evaporation fractionation leads to changes in hydrogen and oxygen isotope composition in the remaining water body. The water absorption and fermentation processes of plants record the fingerprint of the water at the production area, while the distillation process may result in the difference between the hydrogen and oxygen isotope composition of alcohol and raw water due to physical fractionation [19,20]. Also, ^87^Sr/^86^Sr, which is mainly derived from the radioactive decay of rubidium (Rb) in various geological samples, and the ^87^Sr/^86^Sr in alcoholic beverages can provide geographical information, as strontium originates from the soil and water sources of geographical origin. These stable isotope fingerprints are pivotal for origin traceability, vintage identification, and detecting adulteration across a wide range of alcoholic beverages.

This review consolidates recent advancements in stable isotope techniques for alcoholic beverage authenticity, focusing on origin traceability, fraud detection, and vintage identification. It also proposes forward-looking strategies, including the integration of stable isotope data with machine learning models, the creation of a global isotopic database, and the exploration of novel biomarkers. By addressing these gaps, this work aims to provide a comprehensive framework for enhancing the reliability and applicability of stable isotope methods in the global beverage market.

## 2. Origin Traceability

The geographic origin of alcoholic beverages significantly influences their quality, taste, and market value. Factors such as plant ingredients, water sources, microorganisms, climate, and local brewing traditions contribute to the distinct characteristics of beverages produced in specific regions. These factors also combine to create unique stable isotope fingerprints that reflect geographic origin and environmental conditions. Stable isotope analysis has emerged as a powerful tool to trace the geographical origin of alcoholic beverages, offering a scientific basis for authenticity verification and helping combat fraud and mislabeling in the global beverage market.

**Figure 1:**
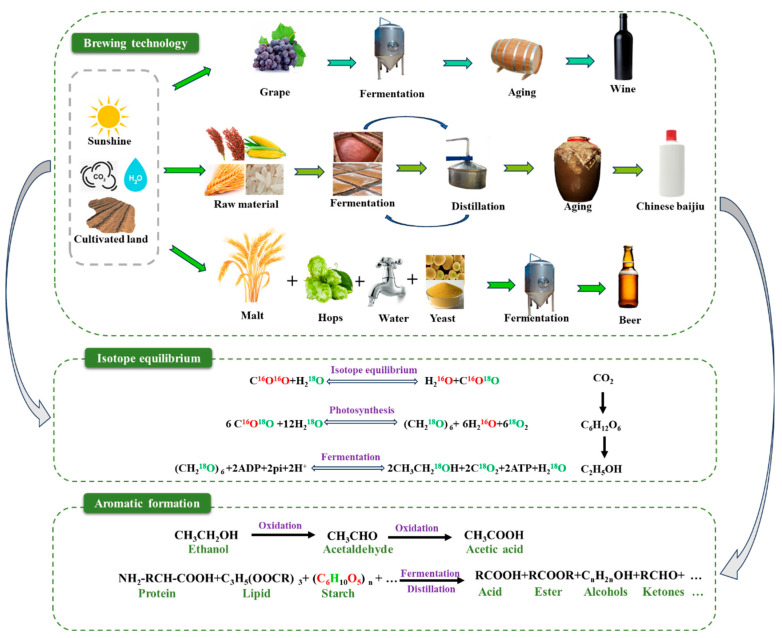
Brewing process of different beverages, formation of aromatic substances, and fractionation of stable isotopes.

The ingredients for the production of alcoholic beverages are primarily derived from plants (such as grapes, sorghum, corn, malt, etc.), which produce ethanol and various flavor compounds through fermentation by microorganisms (primarily yeast) [21,22]. Figure 1 illustrates the brewing process of different varieties of alcoholic beverages and the fractionation effect of isotopes in different processes. In detail, plant ingredients synthesize organic compounds (e.g., proteins, lipids, starch, sugars, etc.) through photosynthesis during growth, and then these organic compounds ferment to produce ethanol and carbon dioxide under the action of yeast during the brewing of alcoholic beverages [22]. Stable isotopes effectively indicate the types of plants, water sources, and production processes involved in the production of alcoholic beverages [23]. Therefore, the stable isotopes of the plants, water, and other flavor components together form the isotopic fingerprint of the geographical origin of the alcoholic beverages.

### 2.1. Wine

Wine is an economic agricultural product whose commercial value is highly dependent on geographic location, grape variety, and vintage [24]. Wine vinification is a complex biological process, and its carbon, oxygen, and hydrogen isotopic composition is closely related to grape variety, cultivation practices, vinification process, and growing environment [25], and carries information about local soil, climate, wine processing, and storage [8,26].

Stable isotopes such as hydrogen (δ^2^H), oxygen (*δ*^18^O), carbon (*δ*^13^C), and strontium (^87^Sr/^86^Sr) play critical roles in identifying wine geographical origin. The isotopic signature of wine reflects the climatic and environmental conditions of the vineyard, including soil composition, temperature, precipitation, and altitude. Soil composition, particularly the presence of minerals and organic matter, influences the isotopic ratios of elements in grapes. Recent studies have shown that the isotopic ratios of nitrogen (δ^15^N) and carbon (δ^13^C) in grapes are significantly affected by the type of fertilization used during cultivation [27]. For example, organic and biodynamic cultivation systems produce distinct isotopic signatures compared to conventional systems, with organic grapes showing higher δ^15^N and δ^13^C values [27]. Additionally, the isotopic composition of soil nitrogen can vary based on land use history and environmental conditions, further influencing the isotopic markers in grapes [28]. These findings highlight the importance of soil composition in shaping the isotopic fingerprints of grapes and, consequently, wine. For example, the *δ*^13^C values of ethanol in Romanian wine are −27.4 ± 1.0‰ [29], −28.7 ± 0.9‰ in Japanese wine [30], −28.2 ± 0.9‰ in Austrian wine [2], −28.4 ± 0.9‰ in Swiss wine [31], and −28.4 ± 0.8‰ in Slovenian wine [2]. The *δ*^18^O value of water in wine exhibits a strong correlation with the spatial climatic and precipitation *δ*^18^O patterns associated with grape harvest [17,32,33]. This relationship underscores the potential of *δ*^18^O as a reliable indicator for determining the geographic origin of wine. The *δ*^18^O values in wines from different countries and geographical origins vary significantly. For example, the *δ*^18^O of water in wine is 1.4 ± 2.6‰ in Croatia [34], 7.8 ± 1.6‰ in Cyprus [35], and −2.6 ± 1.1‰ in Japan [36]. The value of *δ*^18^O is highly dependent on the climate and location, primarily latitude, temperature, and precipitation [37]. In addition, as latitude, altitude, and distance from the ocean increase, the *δ*^18^O value in precipitation generally decreases, thus affecting the *δ*^18^O value in wine [16]. Su et al. [38] investigated the spatial distribution of oxygen isotopes in Chinese wines; the results showed that the *δ*^18^O in wine water becomes more negative with increasing altitude, the *δ*^18^O values of wine in Xinjiang (2.5 ± 2.5‰) > Helan Mountains (−0.3 ± 1.9‰) > Loess Plateau (−2.7 ± 3.7‰) > Southwest Mountains (−7.2 ± 2.9‰), while their average elevations show an opposite trend as Xinjiang < Helan Mountains < Loess Plateau < Southwest Mountains. Meanwhile, their study also demonstrated that the *δ*^18^O values of wine were more negative in the western part of Xinjiang than in the eastern part, likely due to the greater distance from the ocean in the west.

Similarly, strontium isotopes (^87^Sr/^86^Sr) have proven effective in distinguishing vineyards based on the geological characteristics of their soils [11,39,40], such as those from Italy [41,42], Romania [43], France [39], and China [44]. Epova et al. [39] demonstrated that ^87^Sr/^86^Sr ratios in Bordeaux wines can be used to distinguish vineyards based on their geological characteristics. Recent advancements in integrating isotopic markers with machine learning models, such as random forests and neural networks, have further improved the accuracy of geographic classification for wines [39,40,41,44,45,46]. Therefore, ^87^Sr/^86^Sr serves as an important parameter to characterize the terroir of wines and to distinguish between small or single wine variety vineyards.

Numerous researchers have applied stable isotopes and machine learning methods to determine the geographic origin of wines (Table 1). Based on machine learning, wine can be categorized into three model categories for origin traceability: country [47], state/province [32,48], and village/town [49]. According to the purpose of origin traceability, one or two machine learning methods are selected to establish an origin traceability model to improve discrimination accuracy. Currently, most machine learning methods used in origin traceability research are supervised learning methods, such as Discrimination Analysis (DA), Random Forest (RF), Artificial Neural Network (ANN), and Support Vector Machine (SVM). In practical applications, ANN models demonstrate better traceability accuracy than Partial Least Squares Discrimination Analysis (PLS-DA) models. The ANN also calculates the importance of each variable based on its contribution to the discrimination model [32,47]. ANN methods have higher recognition accuracy and are more suitable for classifying the global geographical origin of wines than Discrimination Analysis or Random Forest methods [38,47]. Random Forest (RF) is generally more suitable for classifying small samples and specific regions, while DA may be more appropriate for complex geographical origin classifications.

### 2.2. Chinese Baijiu

Chinese baijiu is one of China’s most traditional alcoholic beverages. It has a long history of brewing and holds significant cultural value. The brewing characteristics of baijiu are reflected in its diverse craftsmanship, historical origins, regional characteristics, and aromatic richness [21,22]. Accordingly, Chinese baijiu from different regions exhibit significant variations in quality and price [53]. High-quality baijiu production areas often have distinctive characteristics [54]. Thus, research on the origin traceability of Chinese baijiu is crucial to safeguard the reputation of higher-quality production areas and combat adulteration and fraud in the industry.

Existing traceability studies on Chinese baijiu production regions primarily employ inorganic elements [9], organic compound fingerprinting [55], and stable isotope techniques [56]. However, because Chinese baijiu is a distilled spirit [21,22], elemental fingerprints may not fully reflect the raw ingredients and production processes. Furthermore, the complex brewing process for baijiu introduces unpredictable elemental contamination, which may potentially affect elements’ ability to indicate production regions. Moreover, the blending process of baijiu, which is performed by master blenders, leads to variations in organic components (particularly trace organic components) between batches, which significantly impacts the effectiveness of organic compounds as a geographical origin indicator for baijiu [54]. Inorganic elements and volatile compounds are studied at the molecular level to correlate with Chinese baijiu’s geographical origin, but their correlation can be potentially manipulated by adding exogenous substances. In contrast, stable isotope technology examines the atomic level to identify the origin of isotopes in Chinese baijiu, offering the advantage that isotope ratios remain unchanged regardless of the presence or addition of exogenous additives. Thus, stable isotope techniques have greater traceability potential for tracing baijiu production regions.

Stable isotopes effectively indicate the isotopic signatures of plants, water sources, and production processes involved in the production of alcoholic beverages. For instance, C3 plants (such as wheat and barley) typically have δ^13^C values in the range from −34‰ to −22‰, while C4 plants (such as corn and sorghum) have δ^13^C values in the range from −18‰ to −9‰ [15,57]. These differences reflect the distinct photosynthetic pathways used by C3 and C4 plants and are critical for tracing the geographical origin and authenticity of alcoholic beverages containing a variety of cereals. Chinese baijiu is a distilled spirit primarily made from sorghum, corn, and wheat as brewing materials and Daqu or Jiumu as saccharification starter, which is made by steaming, saccharification, fermentation, and distillation [21,22]. Grain from different regions (e.g., sorghum, corn, wheat) exhibit varying isotopic compositions due to factors such as geographic location, soil characteristics, and climatic conditions, and these isotopic signatures are preserved in the Chinese baijiu. Researchers have confirmed a correlation between carbon isotope ratios in Chinese baijiu’s raw ingredients and their geographical origin using stable isotope ratio mass spectrometry (IRMS). For example, the *δ*^13^C values of ethanol in Chinese baijiu from Shandong Province ranged from −14.4‰ to −12.7‰, and Shanxi Province from −10.9‰ to −10.1‰ [56]. Thus, the use of ethanol carbon isotopes can feasibly distinguish Chinese baijiu from these three regions. However, combining other isotopes, organic compounds, and elements is often necessary to more confidently distinguish the geographical origin of Chinese baijiu. Traditional or high-quality baijiu companies typically procure grain and water from specific geographical origins for brewing. Thus, the baijiu’s origin traceability is not strictly based on the actual production site’s environmental and climatic characteristics, but rather reflects the raw ingredients origin used in each brewing company’s production process.

The geographical origin of Chinese baijiu’s ingredients significant impact its quality and flavor, as the climate, soil, water source, and brewing process of ingredients from different regions all significantly affect the final quality and flavor of the Chinese baijiu. However, research on baijiu’s origin traceability remains relatively limited, primarily due to it involving a variety of complex factors, including the source of raw materials, the fermentation process, and storage conditions. To advance this field, additional basic research is necessary, including a systematic study of the isotopic characteristics of baijiu from different regions, establishing a more comprehensive database, and the development of more accurate analytical methods. Furthermore, the combination of chemical analysis, microbiological analysis, and other technical means can provide a more comprehensive scientific basis for tracing the geographical origin of Chinese baijiu.

To enhance origin traceability, it is recommended to combine carbon isotope ratios with additional biomarkers, such as volatile organic compounds and elemental fingerprints, which are recommended. Moreover, the development of a comprehensive isotopic database specific to Chinese baijiu would significantly enhance the reliability of traceability models.

### 2.3. Beer

Beer is the world’s most popular low-alcohol beverage in the world and is produced from water, malt, hops, and yeast [58]. The brewing process of beer is complex and delicate, consisting of several key steps that ensure its unique flavor and quality. Although the geographical origin of beer significantly impacts its taste and quality, the importance of beer origin traceability lies in ensuring product authenticity, protecting geographical indications, safeguarding consumers’ rights and interests, and promoting traditional and newer brewing techniques and culture.

The isotopic signature is primarily influenced by the water source and plant ingredients used in brewing. Research has shown that the isotopic composition of beers varies significantly between regions. For example, beer brewed in Europe primarily uses C3 plants and exhibits lower δ^13^C values than beer from Brazil, where C4 plants such as sugarcane are commonly used as adjuncts. Chesson et al. [59] investigated beer sourced from 33 cities across the United States. The *δ*^2^H and *δ*^18^O distributions ranged from −115‰ to −8‰ and −14.7‰ to −0.7‰, respectively. Hydrogen (*δ*^2^H) and oxygen (*δ*^18^O) reflect the climatic and geographical conditions of the brewing locations. Carter et al. [60] analyzed the *δ*^2^H and *δ*^18^O values of beer and *δ*^13^C values of dried beer residues (mostly sugar) of four beer varieties (ale, lager, stout, or wheat beer) from 162 beers sourced from various global regions. Using Canonical Discriminant Analysis (CDA), they achieved a classification accuracy rate of 68%, which increased to 82% when combined with the 12 elemental markers. However, the limited representation of beers from Africa and Asia, the addition of sugarcane or corn sugar substitutes during brewing, and indiscriminate or falsely labeled products contributed to a lower classification accuracy rate. By analyzing the stable isotope ratios of various elements in beer and using machine learning algorithms for in-depth data analysis and pattern recognition, the geographical origin of beer can be determined more accurately. The application of this technology helps combat fraudulent practices and ensures that consumers receive authentic and reliable product information.

### 2.4. Other Alcoholic Beverages

While stable isotope techniques have been widely applied to wine [32,33], Chinese baijiu [61], and beer [4], their use in tracing other alcoholic beverages, such as sake, fruit spirits, and artisanal liquors, remains underexplored. For instance, isotopic analysis has shown promise in differentiating fruit spirits from closely related regions, despite their similar production processes. Expanding research in this area, particularly in emerging markets and lesser-studied beverages, could uncover new applications and enhance the global impact of stable isotope analysis in authenticity verification. Dehelean et al. [62] analyzed *δ*^13^C, *δ*^2^H, *δ*^18^O, and multi-elements of ethanol in spirit drinks to differentiate between various types of spirits from four regions of Transylvania (Bistrita, Cluj, Salaj, and Satu-Mare). Although the overall identification accuracy reached 64%, this rate may have been influenced by the close geographical proximity and high component similarity in these areas. However, when focusing solely on Bistrita, Cluj, and Satu-Mare, the identification accuracy significantly increased to 91.2%. This result suggests that higher accuracy may be achieved when discriminating samples from regions more geographically dispersed.

For other alcoholic beverages, although there are currently fewer studies at present, it is foreseeable that more studies on tracing their geographical origin alcohols may emerge in the future as technology advances and research deepens. For example, analyzing specific chemical components and isotope ratios in alcoholic beverages, combined with geographic information systems (GIS) and statistical analysis methods, is expected to reveal the geographical origin characteristics of these alcoholic beverages.

### 2.5. General Principles for the Authenticity Assessment of Alcoholic Beverages

**Figure 2:**
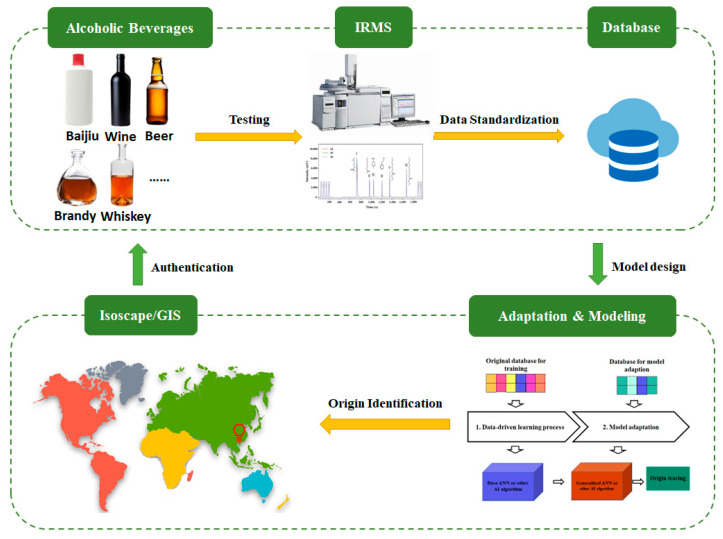
General principles of authenticity assessment of various alcoholic beverages.

The authenticity of alcoholic beverages is assessed using a multi-step approach involving sample collection, scientific analysis (e.g., isotopic and chemical composition), database construction, statistical modeling, and validation against standardized databases (Figure 2). To ensure reliable results, several principles must be followed:

(1) Ensure sample authenticity: Representative and authentic samples are essential for training a robust authenticity model.
(2) Select appropriate stable isotope indicators: Carbon isotopes of ethanol are commonly used to detect ethanol adulteration and ingredient composition. Oxygen isotopes of water can identify origin and water adulteration. Carbon isotopes of ethanol and glycine can differentiate vintage.
(3) Establish a comprehensive database: The database should include information on geographic location, climatic conditions, production date, raw ingredient sources, and brewing processes. Integration with heavy isotopes (e.g., Sr, Pb) and elemental fingerprinting enhance traceability accuracy.
(4) Develop discriminant models: Combine chemometric methods with mass spectrometry data to identify authenticity markers and statistically evaluate traceability models.

These principles provide a robust foundation for ensuring alcoholic beverages’ authenticity, with a model accuracy of 90% or higher considered adequate for national-scale applications [32].

## 3. Adulteration Identification

In April 2009, the U.S. Food and Drug Administration (FDA) formally introduced the concept of Economically Motivated Adulteration (EMA), which categorizes adulteration into six categories based on the type of adulteration [63]. The adulteration of alcoholic beverages, particularly through the addition of exogenous ethanol and unfermented flavoring substances, is illegal and widely regarded as a deceptive practice aimed at reducing production costs, increasing profit margins, or misleading consumers [64]. The main categories of alcohol adulteration include the addition of ethanol, water, or sugar, which poses significant challenges for both regulators and consumers. Below, we delve into these adulteration methods in detail, focusing on their specific applications in alcoholic beverages.

### 3.1. Adulteration with Exogenous Alcohol

#### 3.1.1. Wine

The adulteration of wine is a longstanding issue in the alcoholic beverage market, historically tied to shortages of raw materials and economic pressures. During periods of grape shortages, wineries historically added sugar and alcohol to fermented grape juice to maintain production volumes, a practice that was once legal and widely accepted [17]. Over time, however, some unscrupulous producers began reducing the percentage of pure grape juice in wines, eventually manufacturing fully blended products devoid of any authentic grape juice. These practices, driven by economic motives, raised significant concerns about the quality and authenticity of wines [64]. Today, while wine adulteration persists, its persistence is driven not only by the financial interests of dishonest producers but also by consumers’ limited knowledge and understanding of wine quality.

The adulteration of wine with exogenous ethanol is generally illegal for table wines but may be permitted in the production of liqueur wines under specific regulations [65]. This distinction is important for understanding the context of wine authenticity and the application of isotopic analysis. From a regulatory and analytical perspective, wine adulteration presents a complex challenge that requires the development and application of advanced authentication techniques. Robust and reliable wine authentication is essential to ensure product integrity, protect consumer rights, and foster trust in the global wine market. One innovative approach to detecting wine adulteration involves analyzing of stable isotope ratios of ethanol, glycerol, water, and carbon dioxide. By measuring isotope signatures, it is possible to identify the presence of adulterants such as exogenous sugar, synthetic ethanol, tap water, or industrial carbon dioxide. Perini and Camin [66] analyzed the oxygen isotope ratios (expressed as *δ*^18^O_Eth_) of ethanol in wine and its raw materials. They found significant relationships between the *δ*^18^O_Eth_ values of ethanol and the *δ*^18^O_Water_ values of wine water, establishing *δ*^18^O as a reliable internal check to assure the origin of ethanol, whether derived from grape juice, other fruits or grains, or synthetic products. Their results showed *δ*^18^O values ranging from 24‰ to 36‰ in wine, from 10‰ to 26‰ in fruit and grain distillates, and from −2‰ to 12‰ in synthetic ethanol, thereby effectively distinguishing ethanol origins. Based on the metabolic homology between ethanol and tartaric acid, Zhang et al. [67] established a carbon isotope (*δ*^13^C) detection model to determine whether exogenous C4 alcohol was added to the wine. If the *δ*^13^C difference and the ratio between ethanol and tartaric acid were within −5.2 ± 0.4‰ and 1.2 ± 0.1‰, respectively, it indicated that no exogenous C4 alcohol was added. Exogenous alcohol derived from C4 plants can be detected using carbon isotopes. However, it is not easily detectable, as for exogenous alcohol derived from the C3 plant, because both wine and alcohol derived from the C3 plant belong to C3 photosynthesis, and their carbon isotope ratios are distributed in the range from −34‰ to −22‰ [15,57]. Therefore, in the future, it may be necessary to explore new biomarkers or combine multiple methods to improve the detection of C3 plant sources in alcoholic beverages to ensure the accuracy and reliability of identification results.

#### 3.1.2. Chinese Baijiu

Chinese baijiu can be adulterated through the illegal addition of exogenous alcohol, chemical additives, substitution of non-traditional raw ingredients, and use of non-traditional brewing processes, such as blending with exogenous alcohol before distillation. Detecting baijiu adulteration requires a comprehensive database that describes authentic baijiu manufactured over various years, in different regions, from different raw ingredients. For the carbon stable isotopes in plants, the primary factor influencing the δ^13^C values is the biosynthetic pathway of carbon dioxide fixation. Approximately 85% of plant species follow the C_3_ pathway. C_4_ plants are enriched in ^13^C compared to C_3_ plants [15,57]. The stable carbon isotope ratios of ethanol in Chinese baijiu can reflect the characteristics of its raw materials. Researchers analyzed the ethanol *δ*^13^C values in 33 samples of Jiangxiangxing Chinese baijiu, and two industrially manufactured alcohols’ δ^13^C values ranged from −19.5‰ to −19.1‰ for the baijiu samples and −13.7‰ to −11.6‰ for the industrial alcohols, respectively. These data reveal the significant potential of stable carbon isotope analysis technology in identifying the authenticity of Jiangxiangxing Baijiu, particularly in distinguishing counterfeit Baijiu adulterated with industrial alcohol [68]. In the production process of Chinese baijiu, isobutanol primarily originates from the sugars in the fermentation materials the metabolism of leucine, while ethanol directly comes from the sugars in the raw materials [69]. In Chinese baijiu brewed purely from grains without any external substances addition, the stable carbon isotope ratios of ethanol and isobutanol should theoretically exhibit metabolic consistency. Based on this theoretical foundation, Wang [69] employed isobutanol as an endogenous marker for the stable carbon isotopes of ethanol to identify the presence of exogenously added edible alcohol in Chinese baijiu.

Recently, several innovative adulteration methods for Chinese baijiu have emerged. These include blending exogenous alcohol derived from the fermentation of one or more raw ingredients, where the ethanol *δ*^13^C values are virtually indistinguishable from those of brewed Chinese baijiu. Therefore, relying solely on *δ*^13^C values of ethanol is ineffective in identifying adulteration of exogenous alcohols produced using multiple raw materials. To effectively address this challenge, it is necessary to conduct a more comprehensive characterization of Chinese baijiu using multiple isotopes and elements to build a robust database that can more clearly identify and respond to these emerging adulteration activities.

#### 3.1.3. Other Alcoholic Beverages

Since 2013, the production of ‘Polish Vodka’ has been legally restricted to ethyl alcohol of agricultural origin, exclusively obtained from rye, wheat, barley, oats, triticale, and potatoes grown in the territory of the Republic of Poland. Ciepielowsk et al. [7] accurately determined the deuterium/hydrogen (D/H) ratios at the methyl site (D/H)_I_ and methylene site (D/H)_II_ of ethanol in vodka, as well as the carbon isotope (*δ*^13^C) using NMR and IRMS, making it possible to determine whether Polish vodka is adulterated with cheaper, more readily available corn alcohol. For real alcoholic beverage samples, the internal isotopic correlations between ethanol and congeners from the same sample are significant. Rhodes et al. [70] found that the ^13^C/^12^C ratios of propan-1-ol and ethanol in authentic whiskey samples exhibited a close and reproducible correlation, and the method estimated that the minimum detectable level of neutral alcohol is approximately 20%, depending on the cereal source of the whiskey and neutral alcohol used. Tequila is an alcoholic beverage prepared from the distillation of musts from the *Agave tequilana* Weber blue variety. Researchers established a diagnostic test using gas chromatography-mass spectrometry (GC-IRMS) that can distinguish and identify 100% agave silver, non-authentic beverages, and alcoholic beverages produced from other agave species different from the Weber blue variety of Agave [6,71].

### 3.2. Exogenous Water

The addition of water during the production of alcoholic beverages can affect both product quality and cost efficiency. Plant water isotopic values are typically enriched compared to exogenous water sources. For example, irrigation water sources such as groundwater, river water, and precipitation exhibit different isotopic compositions, with groundwater often being more depleted in δ^18^O and δ^2^H compared to precipitation [27]. Water in plants is more enriched in ^2^H and ^1^⁸O than source water due to plant transpiration [17]. During transpiration, plants are more likely to lose lighter isotopes (^1^H and ^1^⁶O), which leads to enrichment of heavier isotopes (^2^H and ^1^⁸O) in the remaining plant water. When exogenous water is added to an alcoholic beverage, it dilutes the original isotopic signature, leading to depletion of the heavier isotopes in the final product [48]. This change in isotopic composition can be detected through stable isotope analysis, particularly measurements of *δ*^18^O and *δ*^2^H [27], which offers a promising approach for detecting exogenous water in beverages such as wine, baijiu, beer, and Scotch whisky.

#### 3.2.1. Wine

The addition of water to wine reduces the alcohol content and lowers production costs. While this practice is sometimes permitted under specific regulatory frameworks to adjust alcohol content or comply with regional standards, it is generally considered an adulteration practice when used to dilute the wine for economic gain [72]. However, this practice alters the isotopic composition of wine water, particularly the ^18^O/^16^O ratio. Christoph et al. [17] demonstrated the effectiveness of oxygen isotopes in detecting water adulteration in wine. The correlation between *δ*^18^O values of ethanol and wine water provides a diagnostic tool, with deviations indicating dilution.

Recent advancements include mass balance equations [73] and absolute *δ*^18^O differences [74], establishing quantitative thresholds for detecting water adulteration. To ensure wine authenticity, these methods leverage the isotopic discrepancy between fractionated grape water and unfractionated adulterated water.

#### 3.2.2. Chinese Baijiu and Beer

Although isotope-based water detection methods are established for wine, their application to Chinese baijiu and beer remains underexplored. Preliminary evidence suggests that *δ*^18^O and *δ*^2^H isotopes could similarly detect water adulteration in these beverages. Future research should focus on optimization methods, establishing detection limits, and understanding regional isotope variability to confirm feasibility.

#### 3.2.3. Other Alcoholic Beverages

For Scotch whisky, the use of stable isotopes is well documented. Meier-Augenstein et al. [75] highlighted strong correlations between *δ*^2^H and *δ*^18^O values in Scotch water and whisky, enabling differentiation between authentic and counterfeit products. These findings suggest that *δ*^2^H isotopic analyses can monitor not only authenticity but also the geographic origin of water sources. Extending this approach to other spirits, such as rum, gin, and vodka, could enhance global efforts to combat adulteration.

### 3.3. Chaptalization

The addition of sugar, known as chaptalization, is a common practice in the production of alcoholic beverages such as wine, beer, and sake. This process enhances alcohol content and modifies taste during fermentation, but excessive sugar can result in undesirable sweetness and imbalance [58,76]. Stable isotopic analyses, particularly of *δ*^13^C and *δ*^2^H, provide a reliable method for detecting sugar addition, thereby ensuring quality and authenticity.

#### 3.3.1. Wine

Sugar addition in winemaking significantly increases alcohol volume. For every 15.56–18.39 g/L of sugar added, the alcohol content increases by approximately 1% vol [77]. Site-specific natural isotope fractionation nuclear magnetic resonance (SNIF-NMR) technology has been used to trace sugar addition by measuring hydrogen isotopes and alcohol content. This method distinguishes between types of added sugars, such as beet and cane sugar, based on their isotopic signatures [77]. Guyon et al. [78] used HPLC-co-IRMS to simultaneously determine the *δ*^13^C values of glucose (G), fructose (F), glycerol (Gly), and ethanol (Eth), demonstrating that the ratios of these values remain consistent in unadulterated wines. These ratios serve as markers for wine fraud, particularly in cases of sugar addition. Smajlovic et al. [76] introduced *δ*^2^H_n_ in wine ethanol, a parameter indicating non-exchangeable hydrogen isotopes in ethanol. Their research shows that ethanol *δ*^2^H values lower than −217.7‰ indicate sugar or water addition. Combining *δ*^13^C, *δ*^18^O, and *δ*^2^H improves detection sensitivity for adulteration practices, such as sugar addition and must dilution.

#### 3.3.2. Beer

The addition of sugar and starch during beer brewing can increase alcohol content and reduce production costs [58]. Mardegan et al. [79] analyzed the *δ*^13^C values of beers worldwide, correlating isotopic composition with C3 and C4 plant sources. Mardegan et al. [79] used a confidence interval equation proposed by Sleiman et al. [80] and suggested that beers with a confidence interval between −25.9‰ and −26.5‰ contain 100% malt (C3 plants). Conversely, beers with a confidence interval between −18.7‰ and −18.9‰ contain only 50% malt. Their results align with Brazilian legislation Decree No. 2314 (1997) which allows up to 50% malt substitution with C4 sources, such as corn syrup and sugarcane [80]. Voica et al. [81] corroborated this research, finding that beers with *δ*^13^C values less than −25.6‰ indicate the exclusive use of C3 plants in the brewing process, while values greater than −25.6‰ indicate the addition of C4 plant sources. Commonly used supplements include high-maltose corn syrup, corn grits, broken rice, and sugar cane (usually C4 plants) [79].

Carbon isotope ratios serve as strong indicators of adjunct use. European beers, brewed exclusively with C3 plants, exhibit δ^13^C values below −25.6‰, while Brazilian beers, incorporating C4 plants, have higher δ^13^C values [58]. The analysis of δ^13^C can thus quantify sugar sources and ensure compliance with regional brewing standards. Since beer fermentation does not significantly fractionate carbon isotopes, the δ^13^C value of beer is related to the proportion of C3 and C4 components used. Therefore, carbon isotopes can serve as an indicator of whether sugar crops other than malt have been added to beer.

#### 3.3.3. Other Alcoholic Beverages

Sake is a traditional Japanese alcoholic beverage produced through the fermentation of steamed rice and water using koji mold (Aspergillus oryzae) and sake yeast (Saccharomyces cerevisiae) [82]. In sake production, rice starch is converted to glucose via saccharification by hydrolytic enzymes produced by the koji mold [83], and the glucose is converted to ethanol through alcoholic fermentation by sake yeast [84]. Isotopic methods, such as *δ*^13^C and *δ*^2^H analysis, can detect the addition of external sugars or starches. Spiking sake samples with known amounts of glucose, hypothetically from C4 plants, indicate that *δ*^13^C values directly correlate with the amount of exogenous glucose [85]. However, distinguishing glucose from plants of the same photosynthetic type using carbon-stable isotope analysis can be challenging, and other elements, such as hydrogen, must be used to identify the origin of the C3 plant [86]. Expanding isotopic analyses to spirits, such as rum and liqueurs, offers an additional frontier for detecting sugar adulteration. The isotopic markers of sugars from different sources (e.g., cane sugar, and beet sugar) can authenticate product claims and identify fraudulent practices.

### 3.4. Trace Compounds

The trace compounds present in alcoholic beverages shape their complex flavors and contribute to the overall sensory profile of beverages such as wine and Chinese baijiu. These compounds primarily originate from fermentation and microbial metabolism. Recent advances in stable isotope analysis have enabled researchers to detect exogenous additions of trace compounds, thereby safeguarding beverage quality and authenticity. Although alcoholic beverages have been qualitatively and quantitatively analyzed using various methods such as GC-O/MS, GC-MS, GC, and HPLC, studies have not yet determined whether these flavor components are exogenously added. Stable isotopes of trace compounds exhibit metabolic homology relationships [69], allowing for the characterization of trace compounds.

#### 3.4.1. Wine

Wine counterfeiting is a persistent issue [76], often involving the addition of chemical compounds such as tartaric acid and glycerin to mimic authentic wine. It is important to note that the addition of tartaric acid is a legal practice in wine production, regulated to ensure quality and consistency. Tartaric acid is permitted for use in various food categories, including wine, to adjust acidity levels and enhance stability [87]. This practice is regulated under European Union legislation, which allows the addition of tartaric acid to correct natural deficiencies in grapes and to maintain the desired pH balance [87]. Therefore, while the addition of tartaric acid is legal and regulated, it is essential to distinguish between legitimate use and potential adulteration practices that may affect the authenticity of wine; its presence can still be detected through isotopic analysis to ensure that it is sourced from natural grape products rather than synthetic sources [88]. Zhao et al. [89] and Jin et al. [90] developed a carbon isotope detection method for five trace components (e.g., glycerol, isoamyl alcohol) and six volatile organic compounds (e.g., isoamyl acetate, ethyl octanoate). Their findings revealed distinct isotopic differences between authentic and adulterated wines, enabling the detection of exogenous trace components with high accuracy.

#### 3.4.2. Chinese Baijiu

The complex flavor profile of Chinese baijiu arises from a variety of fermentation-derived trace compounds, including organic acids, esters, and alcohols. Stable isotope analysis is instrumental in detecting adulteration in this beverage. Cheng et al. [91] analyzed the *δ*^13^C values of ethyl hexanoate, a key aroma compound, in authentic and adulterated Chinese baijiu. They identified significant isotopic differences, with *δ*^13^C values of adulterated samples ranging from −28.3‰ to −34.0‰ compared to −15.2‰ to −22.1‰ in genuine baijiu. Zhong et al. [92] developed a method to identify exogenously added substances in Chinese baijiu using carbon isotopes. Similarly, Zhang et al. [93] validated isotopic methods for key aroma compounds, such as ethyl hexanoate and hexanoic acid, demonstrating their efficacy in distinguishing authentic brands. These findings highlight the utility of *δ*^13^C values as markers for both authenticity and adulteration in Chinese baijiu. This method demonstrates the effectiveness of using *δ*^13^C values of aroma substances to identify genuine Chinese baijiu brands. Comprehensive knowledge of fermentation pathways and isotopic fractionation is crucial for interpreting stable isotope data in Chinese baijiu. This ensures that authentic flavor profiles are preserved and fraudulent practices are detected.

#### 3.4.3. Other Alcoholic Beverages

Future research should expand isotopic analysis to other alcoholic beverages, such as whiskey, rum, and gin, to develop universal markers for trace compound authenticity. Furthermore, linking isotopic profiles to sensory attributes could enhance consumer understanding of flavor authenticity. Moreover, the integration of advanced analytical technologies, such as IRMS and MC-ICP-MS, holds great promise for improving detection thresholds and precision in the analysis of trace compound isotopes.

## 4. Vintage

Vintage alcoholic beverages, particularly wine and Chinese baijiu, are highly valued for their unique qualities and aging potential. However, counterfeit products undermine market trust, necessitating robust methods to authenticate vintage claims. Stable isotope analysis has proven to be a reliable tool for vintage verification, providing insights into the climatic conditions of the year of production.

### 4.1. Vintage Wine

The vintage year, which reflects the grape harvest, significantly impacts wine flavor, quality, and market value. This correlation arises from the climatic variability that influences grape growth and, consequently, the isotopic composition of the wine. For example, *δ*^13^C values of ethanol are influenced by photosynthetic activity under specific climatic conditions, while *δ*^18^O values of water reflect precipitation and temperature variations [2]. Horacek et al. [2] examined wines from five countries (Austria, Slovenia, Romania, Montenegro, and Argentina) and reported consistent ethanol *δ*^13^C and water *δ*^18^O values across vintages. Despite this consistency, a Linear Discriminant Analysis (LDA) model achieved only a 65% accuracy rate in distinguishing between two vintages. This highlights the need for more advanced techniques, such as combining *δ*^13^C with *δ*^2^H and *δ*^18^O, to enhance vintage classification accuracy. Spangenberg and others have demonstrated that combining isotopic data with sensory or chemical profiles enhances the precision of vintage authentication [2,31]. For example, tannin and polyphenol content, influenced by climatic conditions, could complement isotopic markers to develop a more holistic authentication framework.

Additionally, the *δ*^18^O value correlates closely with the climatic conditions of the harvest year and has been identified as a critical biomarker. Discernible variations in the *δ*^18^O values among wines indicate the distinct climatic conditions encountered throughout the grape growth and harvest season [20]. Notably, years characterized by drought conditions are associated with elevated levels of hydrogen and oxygen isotopes in wine, thereby facilitating the determination of the production year. In a 2016 study by Dinca et al. [29], an examination of 100 wines from four Romania wine regions over the 2009 to 2010 period revealed that the *δ*^18^O values were comparatively lower in 2010 wines across all regions. The reduction was linked to a marked increase in precipitation during the 2010 harvest season. This observation underscores the correlation between the growth year and the oxygen-stable isotope composition of wine water. Furthermore, the study successfully differentiated wines from 2009 and 2010 by employing *δ*^18^O, *δ*^13^C, and a suit of elemental markers (such as chromium, nickel, rubidium, strontium, manganese, lead, copper, zinc, cobalt, and vanadium).

Classifications of wines by vintage substantiate the nexus between wine composition and the unique environmental conditions encountered during the grapes’ maturation and harvest. These findings suggest that variations in climatic and geological conditions during the grape harvest can result in significant disparities among wines from the same production regions and vintage, presenting a challenge for precise vintage identification. Yu et al. [94] collected over 200 wines from the Bordeaux, Burgundy, and Languedoc-Roussillon regions of France, spanning the years 2000 to 2015. They analyzed the *δ*^13^C of wine ethanol and glycine, the *δ*^18^O of wine water, and 16 elements to explore the relationship between climate change and the vintage of French wines using four data-driven models: deep neural networks, decision trees, logistic regression, and support vector machines. Results indicated that isotopic and elemental signatures from various vintages were influenced by precipitation and temperature. When there is less precipitation and higher temperatures during the grape ripening phase, isotopic and elemental signatures have a positive impact on the grapes, resulting in superior-quality French wines. Among these isotopic and elemental fingerprints, *δ*^18^O of wine water, Mg, and Sr exhibited the highest correlation with climatic factors. Data-driven models achieved excellent accuracy in identifying vintages, with identification accuracy reaching up to 72.0%, and even higher accuracy (up to 95.0%) from the Bordeaux region. Consequently, there is an imperative to investigate specific indicators that are minimally influenced by such factors, thereby enhancing the precision of vintage differentiation within the same production regions.

### 4.2. Aged Chinese Baijiu

The concept of aged Chinese baijiu differs from that of wine or foreign spirits. Aged Chinese baijiu typically refers to a percentage of the original baijiu from a specific year being added to the product, rather than the entire bottle being stored for that year [95]. Stable isotope analysis offers a promising avenue for authenticating vintage baijiu. For instance, Cheng et al. [91] analyzed the *δ*^13^C values of ethyl caproate in baijiu of different ages and observed clear isotopic shifts correlated with production years.

Studies conducted by Li et al. [56] and Yue et al. [18] highlight the challenges of identifying aged Chinese baijiu solely on carbon isotopes. These studies found that there was no difference in the *δ*^13^C values of ethanol in different aged Chinese baijiu of the same brand, indicating that ethanol carbon isotopes did not undergo significant fractionation during the storage process. Combining techniques such as gas chromatography-mass spectrometer [95], gas chromatograph-olfactometer [96], gas chromatography-ion mobility spectrometry [97], and radiocarbon (^14^C) analysis [98] with chemometrics is expected to improve the age determination of Chinese baijiu. This multi-method approach may provide a more in-depth assurance of the chemical composition of Chinese baijiu, aiding in the authentication and differentiation of aged Chinese baijiu.

### 4.3. Other Alcoholic Beverages

Beer is typically not labeled with an explicit vintage like wine or Chinese baijiu. Beer is primarily made from barley (or wheat), water, hops, and yeast through fermentation [58]. Although the quality of the raw ingredients affects the quality of the final product, the beer production process is more standardized and generally not limited by seasonal weather conditions. Consequently, the quality of beer tends not to vary significantly from year to year.

Future research should expand vintage authentication methods to other aged alcoholic beverages, such as Scotch whisky, rum, and tequila, where aging significantly influences quality. Furthermore, integrating multi-isotope analysis with machine learning models could enhance classification accuracy. Linking isotopic data with sensory attributes, such as flavor and aroma, would provide a comprehensive authentication framework for vintage beverages. By refining analytical methods and broadening their application, isotopic analysis can play a crucial role in safeguarding the integrity of vintage alcoholic beverages and protecting consumer interests.

## 5. Conclusions and Perspective

Ensuring the authenticity of alcoholic beverages is crucial for protecting consumer trust and safeguarding the industry from counterfeiting and mislabeling. In Europe, stable isotope techniques have been officially adopted to verify wine authenticity, including determining geographical origin, and vintage, and detecting water or sugar adulteration [99,100]. These techniques have demonstrated excellent reliability in practice. However, while isotope techniques for wine are relatively advanced, research on Chinese baijiu, beer, and other alcoholic beverages remains underdeveloped. This is largely due to differences in production processes, raw ingredients, and chemical compositions.

Stable isotope techniques provide a tool for detecting exogenous substances, such as alcohol, water, sugar, and trace ingredients, based on their isotopic distribution patterns. Additionally, isotopic analysis offers insights into the vintage of alcoholic beverages, as climatic conditions vary across years and influence isotope fractionation during plant growth. Future developments in this field are expected to focus on the following areas:

(1) Constructing a comprehensive database: Establish a worldwide stable isotope database of a wide range of alcoholic beverages and regions to facilitate rapid comparative analyses.
(2) Developing novel markers: Identify new indicator molecules that integrate stable isotope data with other chemical and sensory analyses for more accurate authentication.
(3) Advancing analytical technologies: Incorporate emerging technologies such as LC-IRMS, AI-driven isotopic analysis, and IoT-enabled real-time authentication tools.
(4) Promoting interdisciplinary collaboration: Leverage expertise from fields such as ecology, food science, and information technology to drive innovation and application of stable isotope technology.
(5) Addressing practical challenges: Explore ways to reduce the costs of isotopic analysis and improve accessibility for small-scale producers.
(6) Considering climate change impacts: Climate change is emerging as a significant factor that could influence the authenticity and traceability of alcoholic beverages, particularly wine. Rising temperatures, altered precipitation patterns, and increased frequency of extreme weather events are already affecting grape-growing regions worldwide [28]. These changes can lead to shifts in the isotopic signatures of grapes and wines, as stable isotopes are influenced by climatic conditions during plant growth [28]. As climate change progresses, it will be essential to develop new research lines that account for these shifts. This includes updating isotopic databases to reflect changing climatic conditions, exploring novel biomarkers that are less sensitive to climate variability, and integrating isotopic analysis with other tools such as geographic information systems (GIS) and machine learning to enhance traceability and authenticity verification [101].

In conclusion, stable isotope techniques represent a cutting-edge solution for the authenticity assessment of alcoholic beverages, offering reliable methods to verify geographical origin, and vintage, and detect adulteration. With continued advancements in analytical tools, database integration, and interdisciplinary collaboration, these methods promise to deliver safer and higher-quality alcoholic beverages to consumers worldwide. By addressing current challenges and exploring novel directions, the field will continue to evolve, ensuring greater transparency and trust in the global beverage market.

## Figures and Tables

**Table 1:** Wine origin traceability based on stable isotope combined with machine learning (LDA means Linear Discriminant Analysis; ANN means Artificial Neural Network; DA means Discrimination Analysis; SVM means Support Vector Machine; GDA means General Discriminant Analysis; RF means Random Forest; PLS-DA means Partial Least Squares Discrimination Analysis; EA-IRMS means element analysis-isotope ratio mass spectrometer; EQ-IRMS means equilibrium-isotope ratio mass spectrometer; LC-IRMS means liquid chromatography-isotope ratio mass spectrometer; GC-C-IRMS means gas chromatograph-combustion-isotope ratio mass spectrometer; GC-P-IRMS means gas chromatograph-pyrolysis-isotope ratio mass spectrometer).

Model of Origin Traceability	Geographical Origins	Amount	Stable Isotope and Methods	Machine Learning	Accuracy Rate (%)	References
Country	France, Spain, Italy, Australia, the United States, South Africa, Chile, and China	600	*δ*^13^C of glycerol and ethanol: GC-C-IRMS, *δ*^18^O of wine water: Gasbench-IRMS	DA	83.9	[47]
				RF	55.8	
				ANN	93.1	
	Argentina and Austria	62	*δ*^18^O of wine water: Gasbench-IRMS, *δ*^13^C of ethanol: EA-IRMS	LDA	98.4	[50]
	America, Asia, Europe, and Oceania	80	*δ*^18^O of beer water: Gasbench-IRMS, δ^13^C of beer: EA-IRMS, ^87^Sr/^86^Sr of beer: MC-ICP-MS	DA	99	[4]
State/Province	Changji, Mile, and Changli regions in China	188	*δ*^18^O of wine water: GC-P-IRMS	PLS-DA	95	[51]
				SVM	97	
	Hebei Province, Helanshan, Diqing, and Yantai in China	62	*δ*^13^C of glycerol and ethanol: GC-C-IRMS, *δ*^18^O of wine water: Gasbench-IRMS	DA	100	[47]
	Helan Mountain, Xinjiang, Yunchuanzang, Yanhuai Valley, and Hexi Corridor in China	142	*δ*^13^C of glycerol and ethanol: LC-IRMS, *δ*^18^O of wine water: Gasbench-IRMS	LDA	90.8	[33]
	Helan Mountain, Xinjiang, Southwest Mountain, and Loess Plateau regions in China	104	*δ*^18^O of wine water: Gasbench-IRMS	ANN	90.9	[38]
				LDA	88.5	
	Zone B (Croatian Uplands), Zone CI (Slavonia and Croatian Danube), and Zone CII (Croatian Istria and Kvarner) in Croatia	190	*δ*^18^O of wine water: Gasbench-IRMS, *δ*^13^C of wine: EA-IRMS	GDA	86.3	[34]
	Bordeaux, Burgundy, Languedoc-Roussillon and Rhone regions in French	240	*δ*^13^C of glycerol and ethanol: GC-C-IRMS, *δ*^18^O of wine water: Gasbench-IRMS	ANN	98.2	[32]
				PLS-DA	92.5	
	Shanghai, Shaoxing, Xiaogan Beijing in China and Korea	44	*δ*^2^H and *δ*^18^O: Gasbench-IRMS	PLS-DA	97.73	[52]
Village/Town	Three towns (Foshan, Yunling, and Benzilan) Deqin County, plateau of Diqing, Yunnan, China	36	*δ*^13^C of wine: EA-IRMS, *δ*^18^O of wine: EQ-IRMS	DA	80.6	[49]
				ANN	87.7	
	Six counties (YL, BZL, YM, DW, YuL, FS) in Diqing, Yunnan, China	/	*δ*^13^C of wine: EA-IRMS, *δ*^18^O of wine water: Gasbench-IRMS, ^87^Sr/^86^Sr: MC-ICP-MS	DA	100	[44]

## Data Availability

The data presented in this study are available on request from the corresponding author. The data are not publicly available due to privacy restrictions.
